# Lnc-PICSAR contributes to cisplatin resistance by miR-485-5p/REV3L axis in cutaneous squamous cell carcinoma

**DOI:** 10.1515/biol-2020-0049

**Published:** 2020-07-10

**Authors:** Dan Wang, Xiaoqiang Zhou, Jing Yin, Yang Zhou

**Affiliations:** Plastic Surgery Center, Affiliated Hospital of Jiangnan University, Wuxi, Jiangsu, 214000, China; Department of Dermatology, Medical College of Shaoguan University, Shaoguan, 512026, China; Department of Interventional Vascular, Affiliated Hospital of Jiangnan University, Wuxi, Jiangsu, 214000, China; Department of Dermatology, Yancheng First People’s Hospital, Jiangsu Province, No.166 yulong west road, Yancheng, Jiangsu, 224001, China

**Keywords:** CSCC, lnc-PICSAR, miR-485-5p, REV3L, DDP resistance, exosome

## Abstract

**Background:**

Dysregulation of long noncoding RNAs (lncRNAs) is associated with drug resistance in multiple cancers. We explored the roles of lncRNA p38 inhibited cutaneous squamous cell carcinoma-associated lincRNA (PICSAR) in cisplatin (DDP) resistance of cutaneous squamous cell carcinoma (CSCC).

**Methods:**

Quantitative real-time polymerase chain reaction (qRT-PCR) was employed to measure the expression of lnc-PICSAR, miR-485-5p and reversionless 3-like (REV3L) mRNA. The cell counting kit-8 (CCK-8) assay was conducted to evaluate DDP resistance and cell viability. The transwell assay was performed to determine cell migration and invasion. Western blot assay and immunohistochemistry (IHC) staining assay were carried out to measure protein levels. The dual-luciferase reporter assay was used to investigate the association between miR-485-5p and lnc-PICSAR or REV3L. Murine xenograft model was constructed to explore the function of lnc-PICSAR *in vivo*. The morphology of exosomes was analyzed by transmission electron microscopy (TEM) and nanoparticle tracking analysis (NTA).

**Results:**

Lnc-PICSAR was elevated in DDP-resistant CSCC cells. Lnc-PICSAR silencing suppressed cell viability, DDP resistance, migration and invasion in DDP-resistant CSCC cells. MiR-485-5p acted as a target of lnc-PICSAR, and miR-485-5p inhibition reversed the impacts of lnc-PICSAR silencing on DDP resistance and cell progression in DDP-resistant CSCC cells. Lnc-PICSAR promoted REV3L expression via sponging miR-485-5p. Moreover, REV3L overexpression overturned the effects of lnc-PICSAR on cell progression and DDP resistance. Lnc-PICSAR knockdown suppressed DDP resistance *in vivo*. In addition, lnc-PICSAR was increased in the exosomes derived from CSCC patients’ serum and CSCC cells.

**Conclusion:**

Lnc-PICSAR enhanced DDP resistance via miR-485-5p/REV3L axis in DDP-resistant CSCC cells. Besides, exosome-mediated lnc-PICSAR might be involved in the regulation of drug resistance in CSCC.

## 1 Introduction

Cutaneous squamous cell carcinoma (CSCC) is the second most common skin malignancy with high incidence [1]. Because the underlying pathogenesis of CSCC is largely unknown and CSCC has a very strong malignant tendency to metastasize to lymph nodes and other organs, CSCC patients may relapse or even die after treatment [2,3]. Conventional treatment for CSCC patients is the surgery combined with chemotherapy and radiotherapy [4]. Cisplatin (DDP) has been used as an antineoplastic drug in various types of tumors, including CSCC [5]. However, chemotherapy often becomes less effective over time due to the acquisition of drug resistance [6]. To sum up, it is of great significance to explore the underlying mechanism of DDP resistance in CSCC.

Long noncoding RNAs (lncRNAs), a series of noncoding RNAs (ncRNAs) with >200 nucleotides (nts), play crucial roles in cellular processes [7]. Aberrant expression of lncRNAs has been documented to take part in tumor progression and drug resistance. For example, Xue et al. declared that MALAT1 was elevated and facilitated cell growth, metastasis and docetaxel resistance in docetaxel-resistant prostate cancer cells [8]. Shi et al. elucidated that ROR was increased in cisplatin-resistant non-small cell lung cancer (NSCLC) and ROR overexpression enhanced DDP resistance via affecting cell growth, metastasis, autophagy and apoptosis in NSCLC [9]. Zhou et al. manifested that TINCR could modulate ALA-PDL-stimulated apoptosis and autophagy in CSCC [10]. Previous studies demonstrated that p38 inhibited cutaneous squamous cell carcinoma-associated lincRNA (PICSAR) was abnormally expressed and contributed to cell progression in CSCC [11,12]. However, there were no reports on drug resistance of lnc-PICSAR in CSCC.

MicroRNAs (miRNAs), ncRNAs with about 22 nts in length, modulate gene expression via recognizing the 3′-untranslated region (3′UTR) of target mRNAs [13]. Multiple miRNAs are reported to be involved in tumor progression and drug resistance in CSCC. For instance, Xu et al. implicated that miR-125b was reduced in CSCC and served as a tumor suppressor in CSCC by targeting MMP13 [14]. Zhang et al. proved that miR-3619-5p hampered cell growth and DDP resistance in DDP-resistant CSCC cells via interacting with KPNA4 [15]. MiR-485-5p was identified to participate in the modulation of cell progression and chemoresistance in diverse cancers, such as breast cancer [16], oral tongue squamous cell carcinoma (OSCC) [17] and NSCLC [18]. However, the effects of miR-485-5p on CSCC are still unclear.

Reversionless 3-like (REV3L), the catalytic subunit of DNA polymerase ζ, alters the development and chemoresistance of diverse cancers [19]. Wang et al. manifested that REV3L could inhibit the sensitivity of NSCLC cells to DDP [20]. Zhu et al. unraveled that REV3L silencing hampered cell progression and 5-fluorouracil (5-FU) resistance in esophageal squamous cell carcinoma (ESCC) [21]. Nevertheless, the function of REV3L in CSCC and whether miR-485-5p can target REV3L has not been documented.

In this research, we measured the expression of PICSAR, miR-485-5p and REV3L in DDP-resistant CSCC cells. Moreover, their roles and mechanisms in tumor progression and DDP resistance were explored.

## 2 Materials and methods

### 2.1 Serum samples collection

Serum samples were obtained from 30 CSCC patients and 30 healthy volunteers at the Affiliated Hospital of Jiangnan University. The samples were preserved at −80°C until use.


**Informed consent:** Informed consent has been obtained from all individuals included in this study.
**Ethical approval:** The research related to human use has been complied with all the relevant national regulations, institutional policies and in accordance with the tenets of the Helsinki Declaration and has been approved by the Ethics Committee of Affiliated Hospital of Jiangnan University.

### 2.2 Cell culture

Normal human epidermal keratinocytes (NHEKs) and CSCC cells (A431) were bought from the American Type Culture Collection (ATCC, Manassas, VA, USA). CSCC cells (HSC-5) were bought from Japanese Collection of Research Bioresources (Osaka, Japan). DDP-resistant CSCC cells (A431/DDP and HSC-5/DDP) were established by exposing A431 and HSC-5 cells to gradually increased concentrations of DDP (Solarbio, Beijing, China). All cells were grown in Dulbecco’s modified Eagle’s medium (DMEM; HyClone, South Logan, UT, USA) including 10% fetal bovine serum (FBS; HyClone) and 1% penicillin–streptomycin (HyClone) in an incubator containing 5% CO_2_ at 37°C.

### 2.3 Cell transfection

Small-interfering RNA against lnc-PICSAR (si-lnc-PICSAR) and its control (si-NC); the overexpression vector of lnc-PICSAR (lnc-PICSAR) and the overexpression vector of REV3L and their control (pcDNA); miR-485-5p mimic (miR-485-5p) and its control (miR-NC); miR-485-5p inhibitor (anti-miR-485-5p) and its control (anti-miR-NC); and lentiviral vectors (sh-lnc-PICSAR and sh-NC) were purchased from RIBOBIO (Guangzhou, China). Lipofectamine 2000 (Invitrogen, Carlsbad, CA, USA) was utilized for cell transfection.

### 2.4 Quantitative real-time polymerase chain reaction (qRT-PCR)

Total RNA in serums, cells and exosomes was extracted using Rneasy Mini Kit (Qiagen, Valencia, CA, USA). RNAs were reversely transcribed into cDNAs using PrimeScript™ RT reagent Kit (Takara, Dalian, China) or miScript II Reverse Transcriptase Kit (Qiagen). Then, qRT-PCR was conducted with BeyoFast™ SYBR Green qPCR Mix (Beyotime, Shanghai, China). The expression was examined with glyceraldehyde 3-phosphate dehydrogenase (GAPDH) or small nuclear RNA U6 as an internal control using the 2^−ΔΔCt^ method. The primers used were as follows: lnc-PICSAR: (F: 5′-GTGAGCAGAGGGACCTGAAG-3′ and R: 5′-ACATGTGCTCCCCACCTAAG-3′); miR-485-5p: (F: 5′-CCAAGCTTCACCCATTCCTAACAGGAC-3′ and R: 5′-CGGGATCCGTAGGTCAGTTACATGCATC-3′); REV3L: (F: 5′-TGATGTCTTCAGCTGGTATCATGA-3′ and R: 5′-CCGCCCTTCAGGTTCACTT-3′); GAPDH: (F: 5′-GGAGCGAGATCCCTCCAAAAT-3′ and R: 5′-GGCTGTTGTCATACTTCTCATGG-3′); and U6: (F: 5′-TGCGGGTGCTCGCTTCGGCAGC-3′ and R: 5′-CCAGTGCAGGGTCCGAGGT-3′).

### 2.5 Cell counting kit-8 (CCK-8) assay

DDP resistance and cell viability were evaluated by the CCK-8 assay. To determine DDP resistance, A431, A431/DDP, HSC-5 and HSC-5/DDP cells were seeded into 96-well plates and maintained overnight. Then, cells were treated with different doses of DDP (Solarbio) for 48 h. Next, 10 μL CCK-8 (5 mg/mL; Beyotime) was added and incubated for another 2 h. The absorbance at 450 nm was measured using a microplate reader (BioTek, Winooski, VT, USA). The 50% maximal inhibitory concentration (IC_50_) was analyzed.

To determine cell viability, cells were seeded into 96-well plates and incubated overnight. Then, 10 µL CCK-8 (5 mg/mL; Beyotime) was added at indicated time points and incubated for another 2 h. Finally, the absorbance at 450 nm was examined.

### 2.6 Transwell assay

For the analysis of cell migration, A431/DDP and HSC-5/DDP cells in serum-free DMEM (HyClone) were seeded into the upper chamber of a transwell insert (Corning Incorporated, Corning, NY, USA), and DMEM (HyClone) containing 10% FBS (HyClone) was added into the lower chamber. Forty-eight hours later, migrated cells were treated with methanol, stained with crystal violet (Solarbio) and then counted under a light microscope (Olympus, Tokyo, Japan). For the analysis of cell invasion, the transwell chamber was precoated with Matrigel (Solarbio), and the other steps were consistent with the cell migration assay.

### 2.7 Western blot assay

Total protein was isolated from serums, cells and exosomes using RIPA buffer (Beyotime) and quantified using BCA protein assay kit (Tiangen, Beijing, China). Then, equal amount of proteins was separated by sodium dodecyl sulfonate–polyacrylamide gel (Solarbio) and transferred onto polyvinylidene difluoride (PVDF) membranes (Pall Corporation, New York, NYC, USA). After being blocked with skim milk for 2 h, membranes were incubated with primary antibodies against multidrug resistance-associated protein 1 (MRP1; ab32574; Abcam, Cambridge, MA, USA), multidrug resistance protein 1 (MDR1; ab170904; Abcam), REV3L (ab111729; Abcam) or GAPDH (ab181602; Abcam) overnight followed by incubation with corresponding secondary antibody (ab150077; Abcam) for 2 h. The proteins were visualized using the enhanced chemiluminescence detection kit (Vazyme, Nanjing, China).

### 2.8 Dual-luciferase reporter assay

The sequences of lnc-PICSAR and 3′UTR of REV3L, which contained the binding sites of wild-type or mutant miR-485-5p, were inserted into pmirGLO vector (Promega, Fitchburg, WI, USA) to establish luciferase reporter plasmids lnc-PICSAR WT, lnc-PICSAR MUT, REV3L 3′UTR-WT and REV3L 3′UTR-MUT, respectively. A431/DDP and HSC-5/DDP cells were transfected with indicated plasmid and miR-485-5p or miR-NC using Lipofectamine 2000 (Invitrogen). Dual-luciferase reporter assay kit (Promega) was adopted to examine the luciferase activity after 48 h of co-transfection.

### 2.9 Murine xenograft model

Six-week-old nude mice were bought from Shanghai SLAC Laboratory animal Co., Ltd (Shanghai, China) and divided into four groups (*n* = 7). Sh-lnc-PICSAR or sh-NC transfected HSC-5/DDP cells were injected into the nude mice. After 7 days, the mice were treated with 6 mg/kg of DDP (Solarbio) or equivalent phosphate-buffered saline (PBS; Solarbio) every 3 days. Tumor weight was examined every 3 days and calculated with the formula: (length × width^2^)/2. On day 25, mice were sacrificed, and tumor samples were weighed and harvested for subsequent experiments.


**Ethical approval:** The research related to animal use has been complied with all the relevant national regulations and institutional policies for the care and use of animals and has been approved by the Ethics Committee of Animal Research of Affiliated Hospital of Jiangnan University.

### 2.10 Immunohistochemistry (IHC) staining assay

The mice tumors were fixed in 4% paraformaldehyde (Beyotime) for 48 h, embedded in paraffin and sectioned into 4 µm thick. Then, the slides were deparaffinized, hydrated with a graded ethanol series and treated with H_2_O_2_ in methanol for 10 min. Next, the sections were washed with PBS (Solarbio) and incubated with normal goat serum for 20 min. Next, the samples were maintained with anti-REV3L (ab111729; Abcam) at 4°C overnight and HRP-conjugated secondary antibody (ab150077; Abcam) for 30 min at room temperature. After diaminobenzidine (DAB; Beyotime) staining and hematoxylin counterstaining, the sections were photographed using a digital microscope camera (Nikon, Tokyo, Japan).

### 2.11 Isolation of exosomes

Exosomes were isolated from serums using ExoQuick precipitation kit (System Biosciences, Mountain View, CA, USA) based on the instructions of the manufacturer. In brief, serums were centrifuged for 15 min at 3,000 × *g*. Then, the supernatant was mixed with ExoQuick and incubated for 30 min at 4°C and then centrifuged for 30 min at 1,500 × *g*. Next, the supernatant was removed, followed by centrifugation for 5 min at 1,500 × *g* to remove the residual liquid. Exosome pellets were resuspended in PBS (Solarbio). Exosomes from cultured cells were isolated and purified via differential centrifugation as previously described [22].

### 2.12 Transmission electron microscopy (TEM)

Exosomes were placed on carbon-coated copper grids and stained with the phosphotungstic acid solution. The morphology of exosomes was observed by TEM (JEOL Ltd., Tokyo, Japan).

### 2.13 Nanoparticle tracking analysis (NTA)

The size distribution of exosomes was analyzed using Delsa Nano Analyzer (Beckman Coulter, Brea, CA, USA) based on the protocols of the manufacturer.

### 2.14 Statistical analysis

Data were collected from three independent experiments and presented as mean ± standard deviation (SD). Data analysis was conducted using GraphPad Prism 7 software (GraphPad Inc., La Jolla, CA, USA). The difference was analyzed via Student’s *t*-test or one-way analysis of variance (ANOVA). It was considered statistically significant if the *P* value is less than 0.05.

## 3 Results

### 3.1 Lnc-PICSAR was highly expressed in DDP-resistant CSCC cells

**Figure 1:**
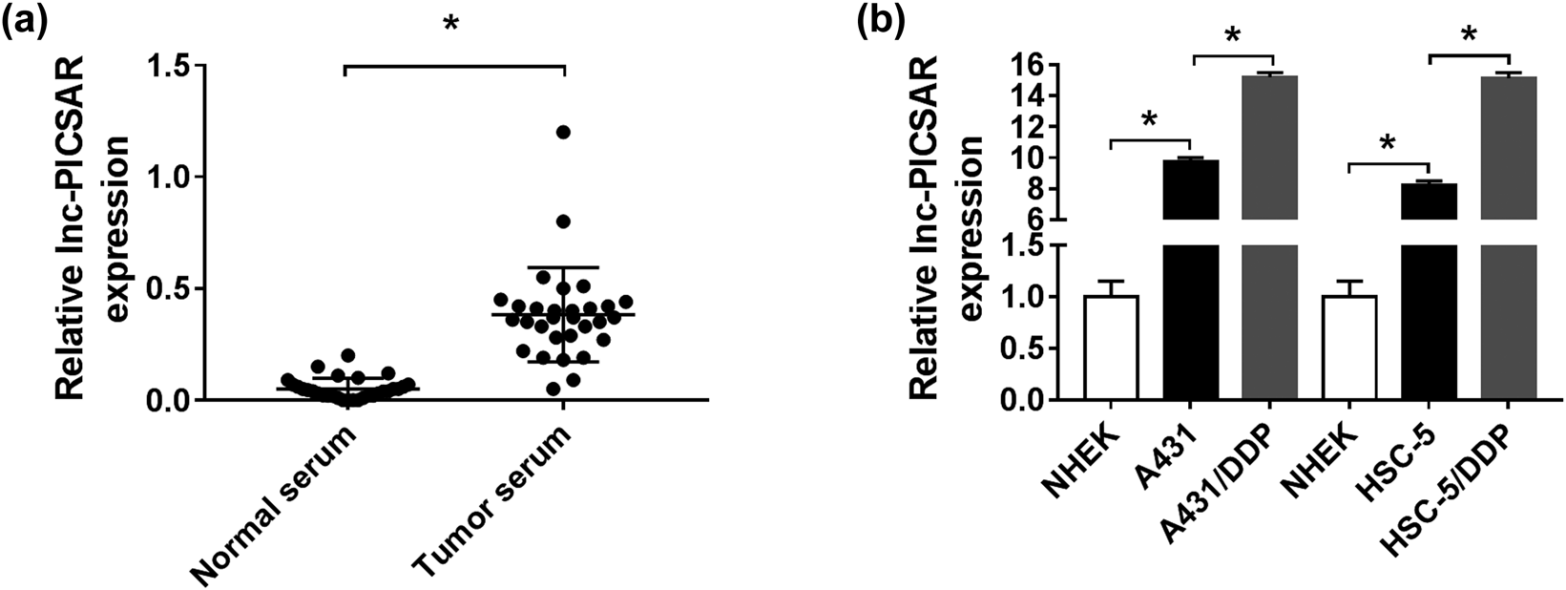
Lnc-PICSAR was upregulated in DDP-resistant CSCC cells. (a) The expression of lnc-PICSAR in the serum of CSCC patients and healthy volunteers was determined by qRT-PCR. (b) The expression of lnc-PICSAR in NHEK, A431, HSC-5, A431/DDP and HSC-5/DDP cells was examined by qRT-PCR. *P < 0.05.

To begin with, we measured the expression level of lnc-PICSAR in the serum of CSCC patients and healthy volunteers by qRT-PCR. The results showed that lnc-PICSAR was conspicuously elevated in CSCC patients’ serum compared to normal serum (Figure 1a). Afterward, lnc-PICSAR expression in NHEK, A431, HSC-5, A431/DDP and HSC-5/DDP cells was examined by qRT-PCR. The data displayed that there was a high expression of lnc-PICSAR in A431 and HSC-5 cells in reference to NHEK cells; moreover, lnc-PICSAR was more highly expressed in A431/DDP and HSC-5/DDP cells compared to that in A431 and HSC-5 cells (Figure 1b). These data indicated that the dysregulation of lnc-PICSAR might be associated with the DDP resistance of CSCC.

### 3.2 DDP-resistant CSCC cells were established

**Figure 2:**
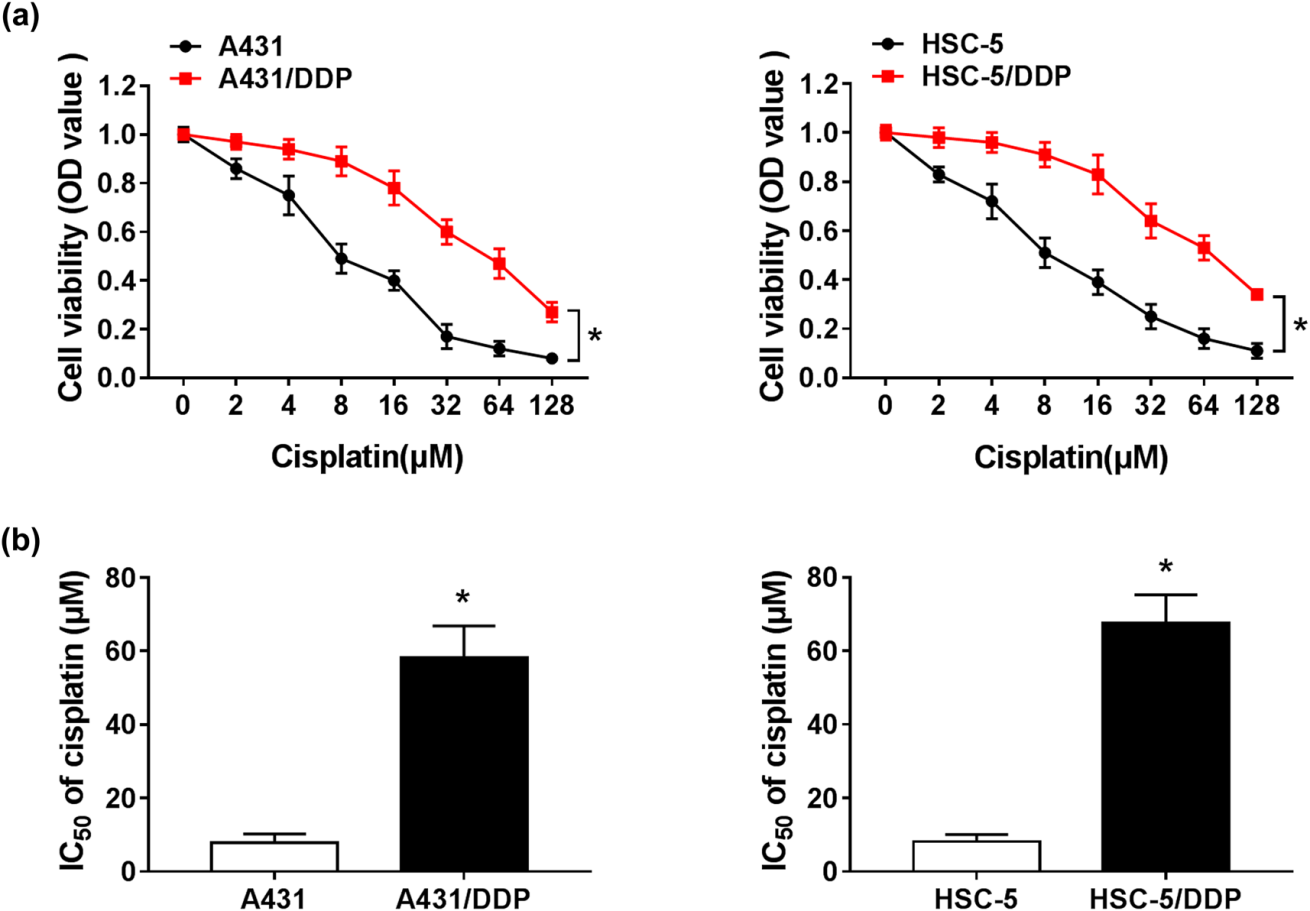
DDP-resistant CSCC cells were constructed. (a) Cell viability of A431, A431/DDP, HSC-5 and HSC-5/DDP cells was determined by the CCK-8 assay. (b) IC_50_ of cisplatin in A431, A431/DDP, HSC-5 and HSC-5/DDP cells was evaluated by the CCK-8 assay. *P < 0.05.

To investigate whether lnc-PICSAR was involved in the DDP resistance of CSCC, we established two DDP-resistant CSCC cells (A431/DDP and HSC-5/DDP). CCK-8 assay indicated that the viability of A431/DDP and HSC-5/DDP cells was enhanced compared to the viability of A431 and HSC-5 cells (Figure 2a). Besides, IC_50_ of cisplatin in A431 cells, HSC-5 cells and corresponding DDP-resistant cells was assessed via the CCK-8 assay. The data manifested that IC_50_ of cisplatin was increased in A431/DDP and HSC-5/DDP cells in reference to A431 and HSC-5 cells (Figure 2b), suggesting that DDP resistance was produced in A431/DDP and HSC-5/DDP cells.

### 3.3 Silencing of lnc-PICSAR suppressed cisplatin resistance in DDP-resistant CSCC cells

**Figure 3:**
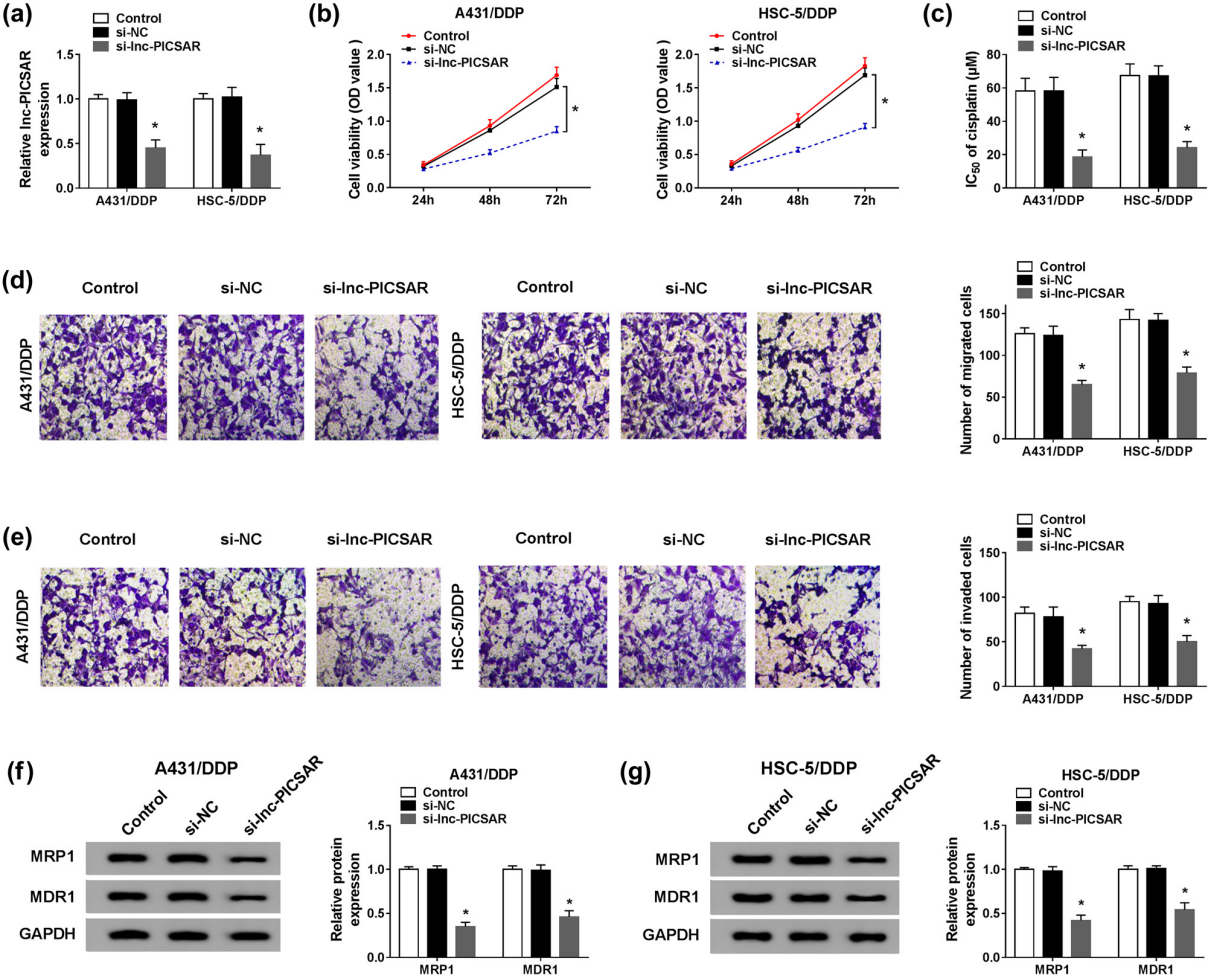
Lnc-PICSAR knockdown enhanced DDP sensitivity in DDP-resistant CSCC cells. A431/DDP and HSC-5/DDP cells were transfected with si-NC or si-lnc-PICSAR. (a) The expression of lnc-PICSAR in A431/DDP and HSC-5/DDP cells was measured by qRT-PCR. (b) The viability of A431/DDP and HSC-5/DDP cells was analyzed by the CCK-8 assay. (c) IC_50_ of cisplatin in A431/DDP and HSC-5/DDP cells was evaluated by the CCK-8 assay. (d and e) The migration and invasion of A431/DDP and HSC-5/DDP cells were assessed by the transwell assay. (f and g) The protein levels of MRP1 and MDR1 in A431/DDP and HSC-5/DDP cells were examined via western blot assay. *P < 0.05.

To explore the exact role of lnc-PICSAR in the resistance of DDP-resistant CSCC cells to DDP, si-lnc-PICSAR was transfected into A431/DDP and HSC-5/DDP cells to downregulate the expression of lnc-PICSAR. As shown in Figure 3a, si-lnc-PICSAR resulted in a remarkable reduction of lnc-PICSAR in A431/DDP and HSC-5/DDP cells compared to the si-NC group. The CCK-8 assay displayed that cell viability was markedly inhibited by lnc-PICSAR silencing in A431/DDP and HSC-5/DDP cells in reference to the si-NC group (Figure 3b). Moreover, the CCK-8 assay showed that IC_50_ of cisplatin was reduced in A431/DDP and HSC-5/DDP cells transfected with si-lnc-PICSAR, indicating that lnc-PICSAR knockdown enhanced the sensitivity of A431/DDP and HSC-5/DDP cells to DDP (Figure 3c). As suggested by the transwell assay, lnc-PICSAR knockdown drastically repressed the migration and invasion of A431/DDP and HSC-5/DDP cells compared to the si-NC group (Figure 3d and e). Besides, the levels of drug resistance-associated proteins were measured via the western blot assay. The data implicated that MRP1 and MDR1 were markedly downregulated in A431/DDP and HSC-5/DDP cells following lnc-PICSAR knockdown (Figure 3f and g). To sum up, lnc-PICSAR knockdown repressed cell viability, DDP resistance, migration and invasion in DDP-resistant CSCC cells.

### 3.4 Lnc-PICSAR knockdown suppressed DDP resistance by targeting miR-485-5p in DDP-resistant CSCC cells

**Figure 4:**
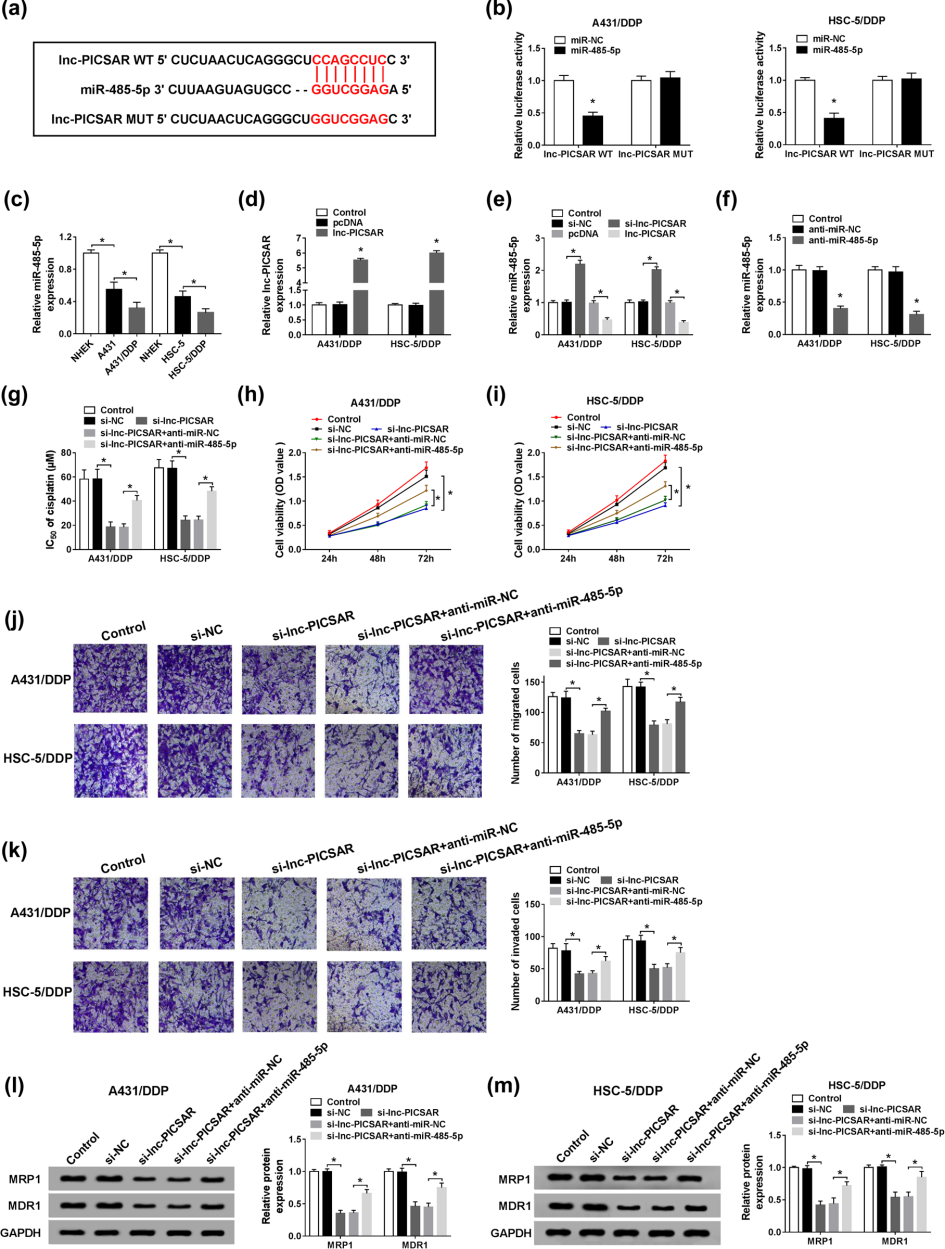
Lnc-PICSAR knockdown repressed DDP resistance via interacting with miR-485-5p in DDP-resistant CSCC cells. (a) The potential binding sites between lnc-PICSAR and miR-485-5p were shown. (b) Dual-luciferase reporter assay was conducted to analyze the association between lnc-PICSAR and miR-485-5p. (c) The expression of miR-485-5p in NHEK, A431, A431/DDP, HSC-5 and HSC-5/DDP cells was measured by qRT-PCR. (d) The expression of lnc-PICSAR in pcDNA or lnc-PICSAR transfected A431/DDP and HSC-5/DDP cells was determined by qRT-PCR. (e) Si-NC, si-lnc-PICSAR, pcDNA or lnc-PICSAR was transfected into A431/DDP and HSC-5/DDP cells, and then, miR-485-5p level was detected via qRT-PCR. (f) Anti-miR-NC or anti-miR-485-5p was transfected into A431/DDP and HSC-5/DDP cells, and then, miR-485-5p expression was measured using qRT-PCR. (g–m) A431/DDP and HSC-5/DDP cells were divided into five groups: control, si-NC, si-lnc-PICSAR, si-lnc-PICSAR + anti-miR-NC and si-lnc-PICSAR + anti-miR-485-5p. (g–i) IC_50_ of cisplatin and cell viability were determined by the CCK-8 assay. (j and k) Cell migration and invasion were analyzed by the transwell assay. (l and m) The protein levels of MRP1 and MDR1 were measured by the western blot assay. *P < 0.05.

To reveal the underlying mechanisms of lnc-PICSAR in the regulation of DDP resistance in DDP-resistant CSCC cells, online website Starbase was used to search the potential target of lnc-PICSAR. As shown in Figure 4a, lnc-PICSAR contained the potential binding sites of miR-485-5p. Then, the dual-luciferase reporter assay was conducted to verify this prediction. The data showed that compared to miR-NC and lnc-PICSAR WT co-transfected cells, the luciferase activity in miR-485-5p and lnc-PICSAR WT co-transfected A431/DDP and HSC-5/DDP cells was distinctly inhibited, whereas the luciferase activity was not affected in lnc-PICSAR MUT groups (Figure 4b). As we expected, miR-485-5p expression was markedly decreased in A431 and HSC-3 cells compared to NHEK cells; moreover, there was lower expression of miR-485-5p in A431/DDP and HSC-5/DDP cells than in A431 and HSC-3 cells (Figure 4c). As shown in Figure 4d and e, lnc-PICSAR transfection caused a significant elevation of lnc-PICSAR and a significant reduction of miR-485-5p, while si-lnc-PICSAR transfection caused a significant increase of miR-485-5p in A431/DDP and HSC-5/DDP cells. These data suggested that lnc-PICSAR decreased miR-485-5p expression by direct targeting in DDP-resistant CSCC cells. We also found that miR-485-5p was decreased in A431/DDP and HSC-5/DDP cells following anti-miR-485-5p transfection (Figure 4f). Subsequently, to reveal whether lnc-PICSAR could alter the resistance of DDP-resistant CSCC cells to DDP, A431/DDP and HSC-5/DDP cells were assigned to control, si-NC, si-lnc-PICSAR, si-lnc-PICSAR + anti-miR-NC and si-lnc-PICSAR + anti-miR-485-5p groups. The CCK-8 assay displayed that IC_50_ of cisplatin was decreased in A431/DDP and HSC-5/DDP cells by lnc-PICSAR deficiency, while inhibition of miR-485-5p partly overturned this effect (Figure 4g). The CCK-8 assay also showed that the inhibitory effect on cell viability mediated by lnc-PICSAR silencing was reversed by miR-485-5p depletion in A431/DDP and HSC-5/DDP cells (Figure 4h and i). Knockdown of lnc-PICSAR resulted in marked suppression in cell migration and invasion in A431/DDP and HSC-5/DDP cells, while the introduction of anti-miR-485-5p weakened the impact (Figure 4j and k). Western blot assay demonstrated that lnc-PICSAR knockdown decreased the levels of MRP1 and MDR1 in A431/DDP and HSC-5/DDP cells, whereas anti-miR-485-5p transfection effectively restored the decrease (Figure 4l and m). All these results indicated that miR-485-5p inhibition reversed the effect of lnc-PICSAR silencing on DDP resistance in DDP-resistant CSCC cells.

### 3.5 MiR-485-5p negatively modulated REV3L expression via targeting REV3L in DDP-resistant CSCC cells

**Figure 5:**
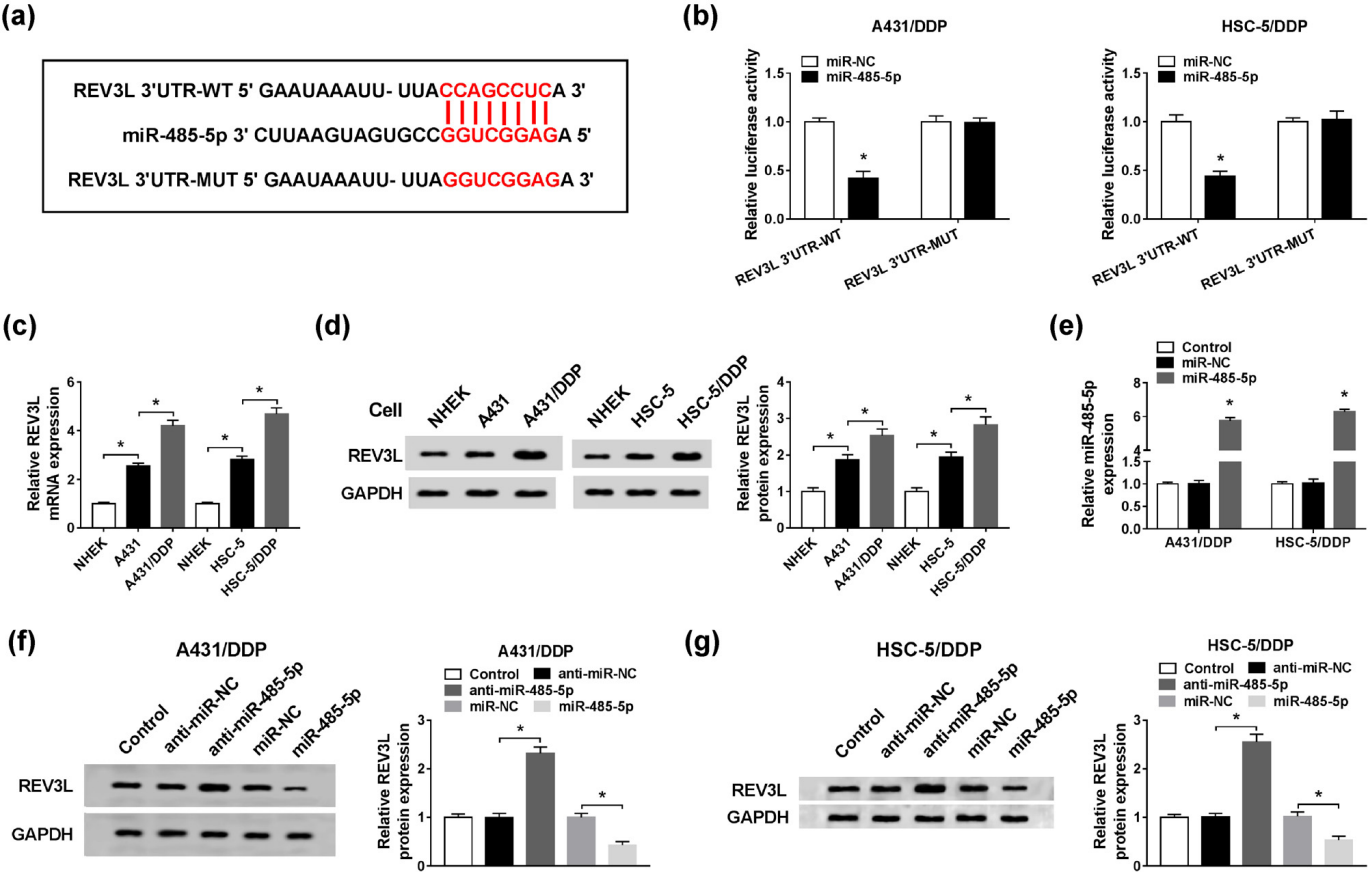
MiR-485-5p directly interacted with REV3L and suppressed REV3L expression in DDP-resistant CSCC cells. (a) The potential binding sites between miR-485-3p and REV3L were predicted by Starbase. (b) The dual-luciferase reporter assay was conducted to determine the association between miR-485-5p and REV3L in A431/DDP and HSC-5/DDP cells. (c and d) The mRNA and protein levels of REV3L in NHEK, A431, A431/DDP, HSC-5 and HSC-5/DDP cells were measured by the qRT-PCR assay and western blot assay, respectively. (e) The expression of miR-485-5p in A431/DDP and HSC-5/DDP cells transfected with miR-NC or miR-485-5p was determined by qRT-PCR. (f and g) A431/DDP and HSC-5/DDP cells were transfected with anti-miR-NC, anti-miR-485-5p, miR-NC or miR-485-5p, and then, the protein level of REV3L was examined by the western blot assay. *P < 0.05.

By using software Starbase, we found that REV3L might be a target gene of miR-485-5p, and their potential binding sites were shown in Figure 5a. Dual-luciferase reporter assay presented that miR-485-5p and REV3L 3′UTR-WT co-transfection led to a remarkable suppression in the luciferase activity in A431/DDP and HSC-5/DDP cells compared to that in miR-NC and REV3L 3′UTR-WT co-transfected cells; however, the luciferase activity was not affected in REV3L 3′UTR-MUT groups (Figure 5b). As displayed in Figure 5c and d, the mRNA and protein levels of REV3L were notably increased in A431 and HSC-5 cells compared to NHEK cells; moreover, the mRNA and protein levels of REV3L were more highly expressed in A431/DDP and HSC-5/DDP cells than in A431 and HSC-5 cells. MiR-485-5p transfection led to an obvious increase of miR-485-5p in A431/DDP and HSC-5/DDP cells (Figure 5e). Next, the effect of miR-485-5p on REV3L expression was determined via transfecting anti-miR-NC, anti-miR-485-5p, miR-NC or miR-485-5p into A431/DDP and HSC-5/DDP cells. The data of western blot assay implied that miR-485-5p depletion promoted REV3L expression, while miR-485-5p overexpression inhibited REV3L expression in A431/DDP and HSC-5/DDP cells (Figure 5f and g). Collectively, miR-485-5p directly targeted REV3L to repress REV3L expression in DDP-resistant CSCC cells.

### 3.6 Lnc-PICSAR knockdown suppressed REV3L expression via miR-485-5p in DDP-resistant CSCC cells

**Figure 6:**
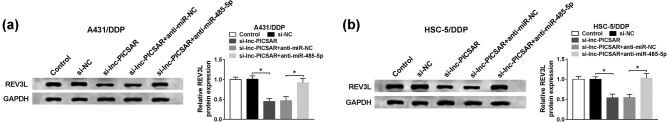
Lnc-PICSAR silencing repressed REV3L expression via miR-485-5p in DDP-resistant CSCC cells. A431/DDP and HSC-5/DDP cells were divided into five groups: control, si-lnc-PICSAR, si-NC, si-lnc-PICSAR + anti-miR-NC or si-lnc-PICSAR + anti-miR-485-5p. (a and b) The protein level of REV3L in A431/DDP and HSC-5/DDP cells was detected using the western blot assay. *P < 0.05.

To further determine the relationship among lnc-PICSAR, miR-485-5p and REV3L in DDP-resistant CSCC cells, A431/DDP and HSC-5/DDP cells were transfected with si-lnc-PICSAR, si-NC, si-lnc-PICSAR + anti-miR-NC or si-lnc-PICSAR + anti-miR-485-5p, and then, the protein level of REV3L was measured. The data displayed that lnc-PICSAR silencing drastically decreased REV3L expression in A431/DDP and HSC-5/DDP cells, but miR-485-5p inhibition restored the effect (Figure 6a and b). The outcomes illustrated that lnc-PICSAR could promote REV3L expression via targeting miR-485-5p in DDP-resistant CSCC cells.

### 3.7 REV3L overexpression weakened the effect of lnc-PICSAR silencing on DDP resistance in DDP-resistant CSCC cells

**Figure 7:**
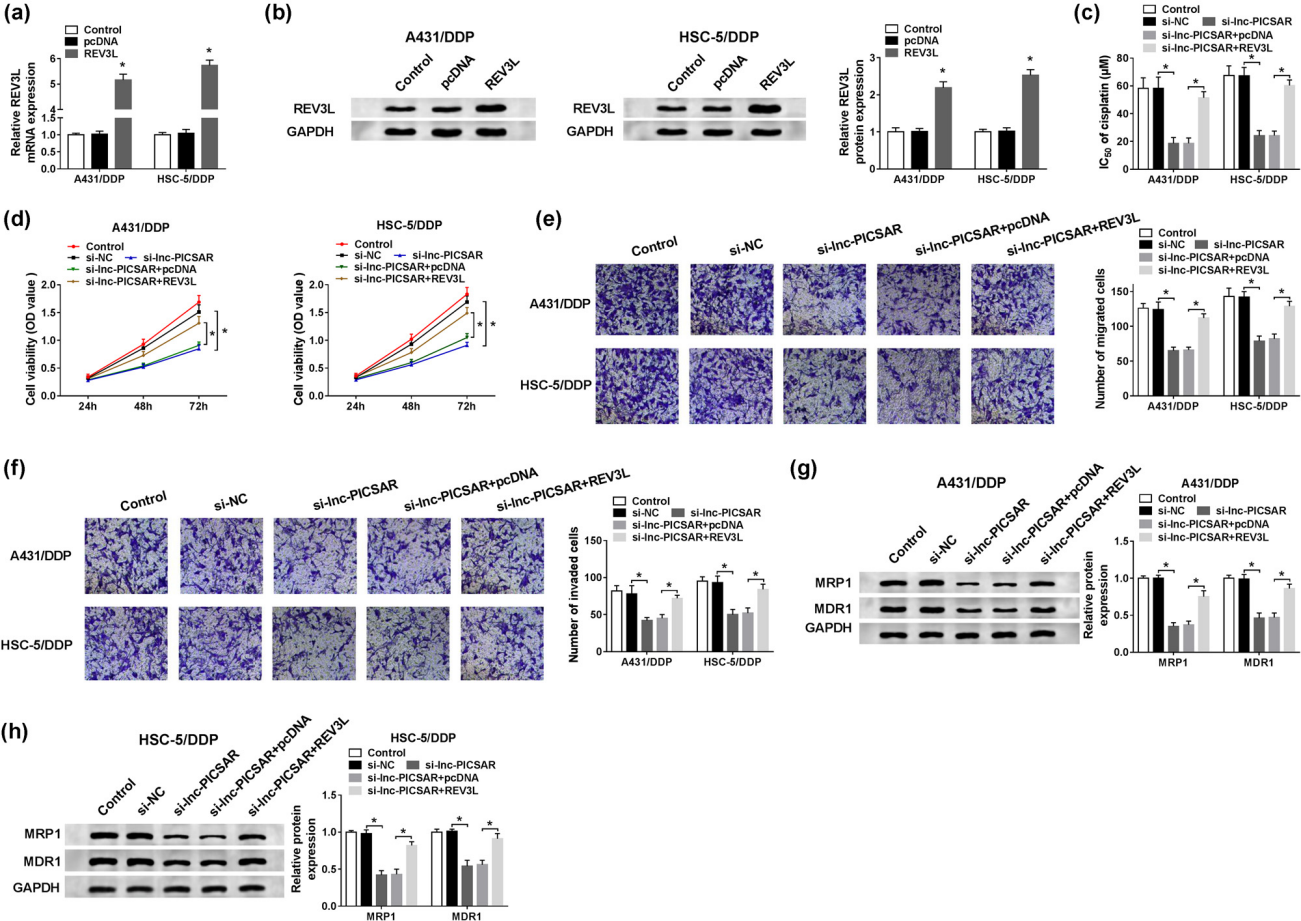
REV3L elevation restored the effect of lnc-PICSAR deficiency on DDP resistance in DDP-resistant CSCC cells. (a and b) The mRNA and protein levels of REV3L in A431/DDP and HSC-5/DDP cells transfected with pcDNA or REV3L were determined by the qRT-PCR assay and western blot assay, respectively. (c–h) A431/DDP and HSC-5/DDP cells were divided into five groups: control, si-NC, si-lnc-PICSAR, si-lnc-PICSAR + pcDNA and si-lnc-PICSAR + REV3L. (c and d) IC_50_ of cisplatin and cell viability in A431/DDP and HSC-5/DDP cells were assessed by the CCK-8 assay. (e and f) The migration and invasion of A431/DDP and HSC-5/DDP cells were examined by the transwell assay. (g and h) The levels of MRP1 and MDR1 in A431/DDP and HSC-5/DDP cells were detected using western blot assay. *P < 0.05.

To further explore the relationship between lnc-PICSAR and REV3L in DDP-resistant CSCC cells, we first determined REV3L level in A431/DDP and HSC-5/DDP cells transfected with pcDNA or REV3L. The data demonstrated that REV3L transfection led to obvious elevation of REV3L mRNA and protein levels in A431/DDP and HSC-5/DDP cells (Figure 7a and b). Afterward, A431/DDP and HSC-5/DDP cells were assigned to control, si-NC, si-lnc-PICSAR, si-lnc-PICSAR + pcDNA and si-lnc-PICSAR + REV3L groups. The CCK-8 assay proved that the inhibitory effects of lnc-PICSAR knockdown on DDP resistance and cell viability were all effectively rescued following the overexpression of REV3L in A431/DDP and HSC-5/DDP cells (Figure 7c and d). The migration and invasion of A431/DDP and HSC-5/DDP cells were hampered by lnc-PICSAR deficiency, while the elevation of REV3L partially ameliorated the effects, as suggested by the transwell assay (Figure 7e and f). Moreover, REV3L overexpression abrogated the suppression on MRP1 and MDR1 levels caused by lnc-PICSAR knockdown in A431/DDP and HSC-5/DDP cells (Figure 7g and h). Taken together, the inhibitory effect of lnc-PICSAR knockdown on DDP resistance was reversed by REV3L in DDP-resistant CSCC cells.

### 3.8 Lnc-PICSAR knockdown suppressed DDP resistance *in vivo*

**Figure 8:**
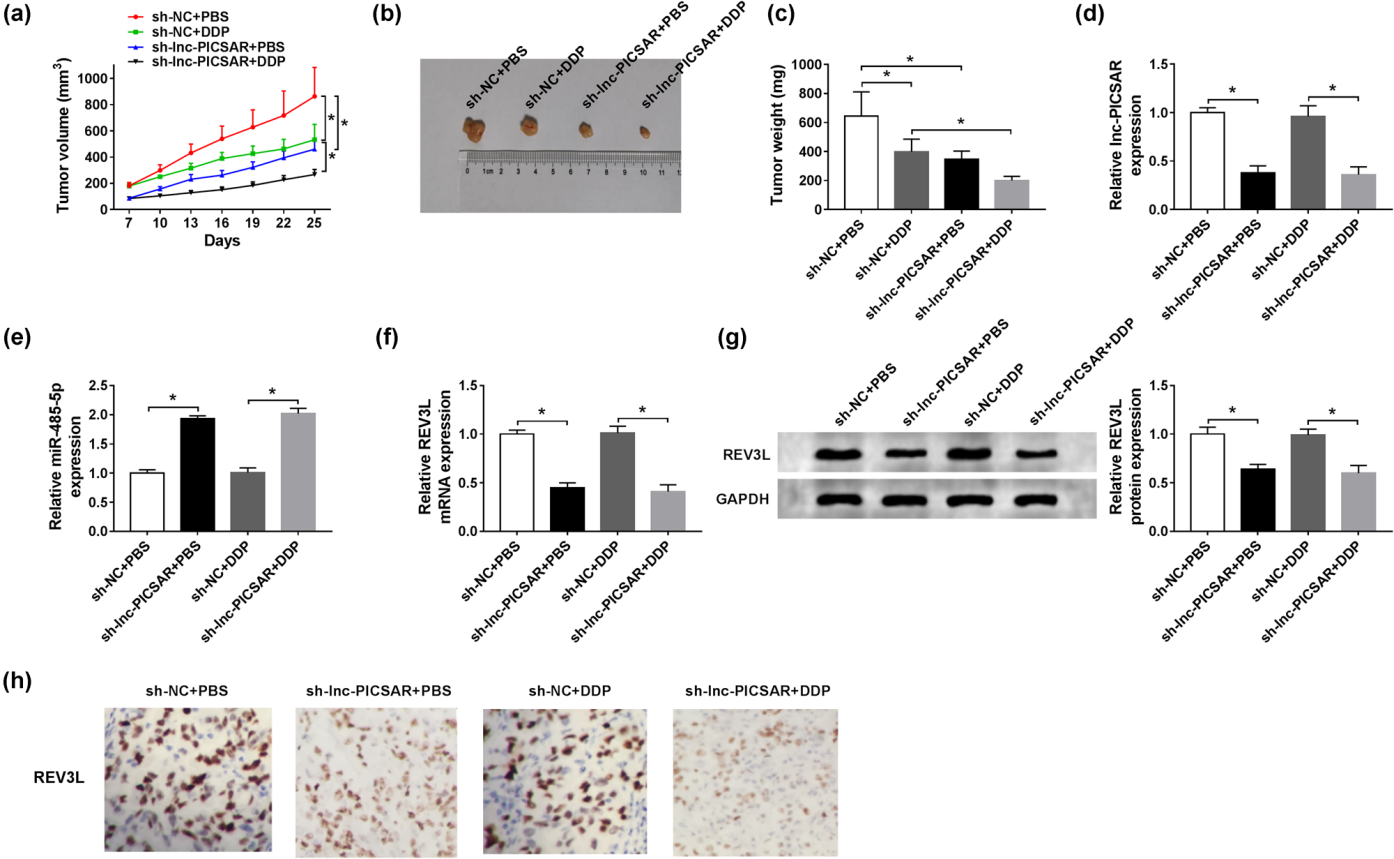
Lnc-PICSAR silencing enhanced the DDP sensitivity of CSCC *in vivo*. HSC-5/DDP cells were transfected with sh-NC or sh-PICSAR and then injected into nude mice. After 7 days, the mice were given 6 mg/kg DDP or equivalent PBS every 3 days. (a) Tumor volume was examined every 3 days. (b and c) Tumor weight was measured after 25 days. (d–f) The expression levels of lnc-PICSAR, miR-485-5p and REV3L mRNA were determined by qRT-PCR. (g) The protein level of REV3L was detected by the western blot assay. (h) IHC staining of mice tumor tissues to evaluate the level of REV3L. *P < 0.05.

To reveal the effect of lnc-PICSAR on DDP resistance *in vivo*, we established a xenograft tumor mouse model by injecting sh-NC or sh-PICSAR transfected HSC-5/DDP cells into nude mice. Seven days later, the mice were treated with 6 mg/kg DDP or equivalent PBS every 3 days. We observed that tumor volume and tumor weight were markedly blocked by the DDP treatment or lnc-PICSAR knockdown (Figure 8a–c). Moreover, the levels of lnc-PICSAR, REV3L mRNA and REV3L protein were drastically reduced, and miR-485-5p was drastically increased in tumor tissues from sh-lnc-PICSAR groups compared to sh-NC groups, as analyzed by the qRT-PCR assay, western blot assay and IHC assay (Figure 8d–h). We concluded that lnc-PICSAR silencing could improve DDP sensitivity of CSCC *in vivo*.

### 3.9 Lnc-PICSAR was increased in the exosomes derived from CSCC patients’ serum and HSC-5 cells

**Figure 9:**
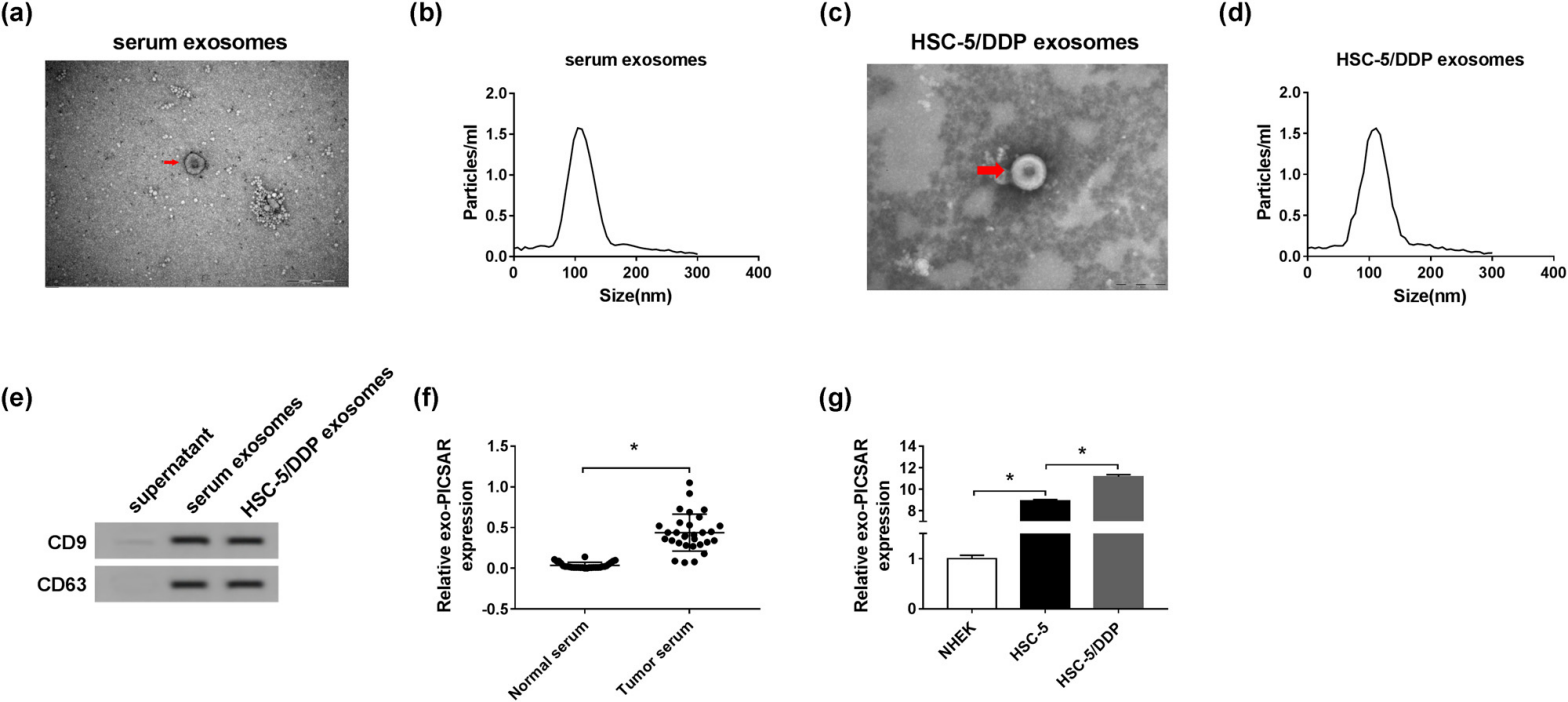
Upregulation of lnc-PICSAR in exosomes derived from CSCC patients’ serum and HSC-5 cells. (a and c) Exosome micrograph was observed by TEM. (b and d) The size distribution of exosomes was analyzed by NTA. (e) Exosomal markers (CD9 and CD63) derived from CSCC patients’ serum and HSC-5 cells were measured by the western blot assay. (f and g) The expression of lnc-PICSAR in the exosomes from serum and cells was detected by qRT-PCR. *P < 0.05.

Subsequently, CSCC patients’ serum-derived exosomes and HSC-5 cells–derived exosomes were isolated. The morphology of exosomes was observed by TEM, and the results showed that vesicles had a round or oval membrane (Figure 9a and c). The NTA assay showed that the size of these nanoparticles was 80–140 nm (Figure 9b and d). Western blot assay showed that the markers of exosomes (CD9 and CD63) could be detected in the exosomes derived from CSCC patients’ serum and HSC-5 cells (Figure 9e). Furthermore, we determined the level of lnc-PICSAR in the exosomes via the qRT-PCR assay. As displayed in Figure 9f, lnc-PICSAR was elevated in the exosomes derived from CSCC patients’ serum. In addition, lnc-PICSAR expression in the exosomes from HSC-5 cells was higher than that in the exosomes from NHEK cells; moreover, lnc-PICSAR was highly expressed in the exosomes from HSC-5/DDP cells compared to that in the exosomes from HSC-5 cells (Figure 9g). These data indicated that lnc-PICSAR could be carried by the exosomes from CSCC patients’ serum and CSCC cells.

## 4 Discussion

Chemoresistance is an enormous obstacle for the therapy of human cancers. LncRNAs have been demonstrated to act as vital regulators in tumor progression and drug resistance in cancers. However, the effects of lncRNAs in chemoresistance of CSCC have been limited to date. Herein, we explored the roles of lnc-PICSAR in DDP resistance in CSCC. We found that lnc-PICSAR was drastically increased in DDP-resistant CSCC cells. Silencing of lnc-PICSAR enhanced the DDP sensitivity via suppressing cell viability, migration and invasion in DDP-resistant CSCC cells. Moreover, we established a novel network of lnc-PICSAR/miR-485-5p/REV3L in the drug resistance of CSCC.

Piipponen et al. validated that lnc-PICSAR was elevated in CSCC cells, and its deficiency hampered CSCC cell growth and migration *in vitro* and blocked the tumor growth *in vivo* [12,23]. Multiple lncRNAs participate in the regulation of human cancers. For example, Bian et al. unfolded that UCA1 improved the resistance of colorectal cancer cells to 5-FU [24]. Li et al. verified that HOTTIP contributed to cell progression and gemcitabine resistance in pancreatic cancer [25]. We investigated the effect of lnc-PICSAR in the resistance of CSCC cells to DDP for the first time. Lnc-PICSAR was markedly elevated in CSCC patients’ serum and CSCC cells. Moreover, lnc-PICSAR expression in DDP-resistant CSCC cells was higher than in DDP-sensitive CSCC cells. Deficiency of lnc-PICSAR improved DDP sensitivity and hampered cell viability and metastasis in DDP-resistant CSCC cells. Moreover, lnc-PICSAR knockdown could inhibit DDP resistance and tumor growth *in vivo*. All these observations suggested that the level of lnc-PICSAR might be a prognostic marker, and lnc-PICSAR enhanced DDP resistance in DDP-resistant CSCC cells.

LncRNAs can function as miRNA sponges to alter the biological function of miRNAs [26]. We wondered whether lnc-PICSAR could serve as a miRNA sponge. We found that lnc-PICSAR acted as a sponge of miR-485-5p, and miR-485-5p was dropped in DDP-resistant CSCC cells. Furthermore, miR-485-5p inhibition weakened the inhibitory effects of lnc-PICSAR deficiency on cell viability, metastasis and drug resistance in DDP-resistant CSCC cells. Wang et al. reported that miR-485-5p could target survivin to repress tumor growth and chemoresistance in breast cancer [16]. Lin et al. manifested that miR-485-5p hampered OSCC cell metastasis and enhanced the sensitivity of OSCC cells to DDP via targeting PAK1 [17]. Our data were in line with the studies by Wang et al. and Lin et al. In addition, REV3L was a target gene of miR-485-5p and its expression was negatively modulated by miR-485-5p in DDP-resistant CSCC cells. REV3L has been demonstrated to mediate tumor development and chemosensitivity in diverse cancers, such as NSCLC [20], ESCC [21,27] and squamous cell carcinoma of the head and neck [28]. In the current study, the elevation of REV3L restored the effects of lnc-PICSAR silencing on cell progression and DDP resistance in DDP-resistant CSCC cells, indicating that REV3L facilitated the progression of DDP resistance in CSCC.

Exosomes are tiny vesicles about 40–150 nm in diameter secreted by cells [29]. It has been documented that exosomes secreted from tumor cells can affect the development of tumors and drug resistance via the transfer of ncRNAs. For example, Deng et al. implicated that the exosomes derived from mesenchymal stromal cells facilitated the cell growth and hampered cell apoptosis in multiple myeloma by linc00461 [30]. Zhang et al. validated that lncRNARP11-838N2.4 was elevated in the exosomes derived from erlotinib-resistant NSCLC patients’ serum and cells and enhanced erlotinib resistance in NSCLC [31]. We found lnc-PICSAR was increased in the exosomes from CSCC patients’ serum and CSCC cells compared to normal serum and cells; moreover, lnc-PICSAR expression was higher in DDP-resistant CSCC cells than in DDP-sensitive CSCC cells. These data indicated that exosome-transmitted lnc-PICSAR might take part in the regulation of chemoresistance in CSCC and we will verify this in the future.

In conclusion, lnc-PICSAR was highly expressed in DDP-resistant CSCC patients’ serum and cells. Lnc-PICSAR/miR-485-5p/REV3L axis participated in the regulation of DDP resistance via altering cell viability and metastasis. These findings suggested that lnc-PICSAR might be a diagnostic biomarker for CSCC and provide a new treatment strategy for CSCC patients.
